# Molecular Characterization of Copper and Cadmium Resistance Determinants in the Biomining Thermoacidophilic Archaeon *Sulfolobus metallicus*

**DOI:** 10.1155/2013/289236

**Published:** 2013-02-24

**Authors:** Alvaro Orell, Francisco Remonsellez, Rafaela Arancibia, Carlos A. Jerez

**Affiliations:** ^1^Laboratory of Molecular Microbiology and Biotechnology, Department of Biology and Millennium Institute for Cell Dynamics and Biotechnology, Faculty of Sciences, University of Chile, Santiago, Chile; ^2^Department of Chemical Engineering, North Catholic University, Antofagasta, Chile

## Abstract

*Sulfolobus metallicus* is a thermoacidophilic crenarchaeon used in high-temperature bioleaching processes that is able to grow under stressing conditions such as high concentrations of heavy metals. Nevertheless, the genetic and biochemical mechanisms responsible for heavy metal resistance in *S. metallicus* remain uncharacterized. Proteomic analysis of *S. metallicus* cells exposed to 100 mM Cu revealed that 18 out of 30 upregulated proteins are related to the production and conversion of energy, amino acids biosynthesis, and stress responses. Ten of these last proteins were also up-regulated in *S. metallicus* treated in the presence of 1 mM Cd suggesting that at least in part, a common general response to these two heavy metals. The *S. metallicus* genome contained two complete * cop* gene clusters, each encoding a metallochaperone (CopM), a Cu-exporting ATPase (CopA), and a transcriptional regulator (CopT). Transcriptional expression analysis revealed that *copM* and *copA* from each *cop* gene cluster were cotranscribed and their transcript levels increased when *S. metallicus* was grown either in the presence of Cu or using chalcopyrite (CuFeS_2_) as oxidizable substrate. This study shows for the first time the presence of a duplicated version of the *cop* gene cluster in *Archaea* and characterizes some of the Cu and Cd resistance determinants in a thermophilic archaeon employed for industrial biomining.

## 1. Introduction

Bioleaching is the biological conversion of an insoluble metal compound into a water soluble form [1, 2]. Microbe-based processes have clear economic advantages in the extraction of metals from many low-grade deposits [3], and these metal-extraction processes are usually more environmentally friendly than physical-chemical processes [3–5]. Some ores are refractory to mesophilic leaching and temperatures preferably as high as 75–85°C are required [6, 7]. At high temperatures, biomining consortia are dominated by thermoacidophilic *Archaea* from the genus *Sulfolobus*, *Acidianus,* and *Metallosphaera* [8]. 

Metals play an integral role in the life process of microorganisms, but at high levels both essential and nonessential metals can damage cell membranes, alter enzyme specificity, disrupt cellular functions, and damage the structure of DNA [9, 10]. Acid-leaching solutions are characterized by high metal concentrations that are toxic to most life, and as might be expected, microorganisms that grow in mineral-rich environments are, in most cases, remarkably tolerant to a wide range of metal ions [3, 11] and should possess robust metal resistance mechanisms [11–15]. 

Despite this, only some metal tolerance values have been reported [6] and the genetic and biochemical mechanisms responsible for metal resistance in biomining acidophilic *Archaea* are just beginning to be characterized [16]. It is therefore important to further understand the mechanisms used by these microorganisms to adapt to and to resist high concentrations of heavy metals.

Related to archaeal copper (Cu) resistance mechanisms, a few metal efflux pumps have been identified from sequenced genomes of some members of this domain [17]. A Cu-resistance (*cop*) locus has been described in *Archaea,* which includes genes encoding a new type of archaeal transcriptional regulator (*CopT*), a putative metal-binding chaperone (*CopM*), and a putative Cu-transporting P-type ATPase (*CopA*) [10]. The same Cu-resistance mechanism was described in *Sulfolobus solfataricus* P2 and *Ferroplasma acidarmanus*. In both microorganisms, the putative metal chaperones and the ATPase are cotranscribed and their transcriptional levels increase significantly in response to Cu exposure, suggesting that the transport system is operating for Cu efflux [18, 19]. Recently, it was described that the *copRTA* operon from *S. solfataricus* strain 98/2 (*copTMA* in *S. solfataricus* P2) is cotranscribed at low levels from the *copR* promoter under all conditions, whereas increased transcription from the *copTA* promoter took place in the presence of Cu excess. These authors proposed a model for Cu homeostasis in *Sulfolobus* which relies on Cu efflux and sequestration [20].

In* silico* studies have further identified a CPx-ATPase which most likely mediates the efflux of heavy metal cations in the biomining archaeon *Metallosphaera sedula* [21]. This putative protein has significant identity to a P-type ATPase from *S. solfataricus *(CopA) [19]. Moreover, *M. sedula *contains ORFs with significant similarity to both *CopM* (Msed0491) and *CopT* (Msed0492) from *S. solfataricus* [21]. Very recently, Maezato et al. [22] have reported a genetic approach to investigate the specific relationship between metal resistance and lithoautotrophy during biotransformation of chalcopyrite by *M. sedula*. The functional role of its *copRTA* operon was demonstrated by cross-species complementation of a Cu-sensitive *S. solfataricus copR* mutant [22].

Cadmium (Cd) is very toxic and probably carcinogenic at low concentrations. However, the biological effects of this metal and the mechanism of its toxicity are not yet clearly understood [23–26]. In some neutrophilic microorganisms, Cd is taken up via the magnesium or manganese uptake systems [23]. Although the mechanisms in acidophiles have not been elucidated, putative Cd resistance operons in some sequenced genomes from acidophilic microorganisms have been identified. The species with the highest homology to the *cadA* motif were *Acidithiobacillus ferrooxidans* and *Thermoplasma* spp. These high similarities suggest that Cd export may be a common resistance mechanism among acidophiles [11]. Recently, a time-dependent transcriptomic analysis using microarrays in the radioresistant archaeon *Thermococcus gammatolerans* cells exposed to Cd showed the induction of genes related to metal homeostasis, drug detoxification, reoxidation of cofactors, ATP production, and DNA repair [27].

One alternative mechanism proposed for metal tolerance in microorganisms is the sequestration of metal cations by inorganic polyphosphates (polyP) [28], and at the same time the intracellular cations concentration would regulate the hydrolysis of this polymer [29]. *S. metallicus* can tolerate very high concentrations of copper and accumulates high amounts of polyP granules [14, 30]. Furthermore, the levels of intracellular polyP are greatly decreased when this archaeon is either grown in 200 mM Cu or shifted to 100 mM Cu [14, 30]. An increase in exopolyphosphatase (PPX) activity and Pi efflux due to the presence of Cu suggests a metal tolerance mechanism mediated through polyP [14, 30]. Actual evidence suggests that polyP may provide mechanistic alternatives in tuning microbial fitness for the adaptation under stressful environmental situations and may be of crucial relevance amongst extremophiles. The genes involved in polyP metabolism in Crenarchaeota have been only partially elucidated, as long as a polyP synthase activity is still to be reported and characterized in this kingdom [15].

 Thus far, there are no studies on the prospective genetic and biochemical mechanisms that enable *S. metallicus *to thrive in such high concentrations of Cu and other metals. Understanding these mechanisms could be particularly useful in potential improvement of the bioleaching microorganisms, which could likely increase the efficiency of bioleaching processes in due course. Since the genome of *S. metallicus* is not currently available, possible genes involved in Cu resistance were searched by using a CODEHOP and “genome walking” approaches and the transcriptional expression of these isolated genes was assessed by real-time RT-PCR. Furthermore, a proteomic approach was used to identify possible proteins involved in resistance to Cu and Cd in *S. metallicus*. To our knowledge, this is the first paper that shows the occurrence of a duplicated version of the *cop* gene cluster in *Archaea* and gives insights into the molecular Cu and Cd resistance determinants in a thermophilic archaeon employed for industrial biomining.

## 2. Materials and Methods

### 2.1. Strains and Growth Conditions

*S. metallicus* DSM 6482 was grown at 65°C in medium 88 (Deutsche Sammlung von Mikroorganismen und Zellkulturen) containing 0.05% (w/v) elemental sulfur and 0.02% (w/v) yeast extract. *S. solfataricus* DSM 1617 was grown at 70°C in medium 182 (Deutsche Sammlung von Mikroorganismen und Zellkulturen) with 0.1% (w/v) yeast extract and with 0.1% (w/v) Casamino acids. To study Cd tolerance of *S. metallicus* and *S. solfataricus*, the microoganisms were grown in their respective media, except that different concentrations of Cd (0.005–5 mM) were present initially, as indicated in the corresponding experiment. 

For differential expression assays, *S. metallicus* cells were grown in the absence of Cu or Cd to the early stationary phase, and after removing the medium from the cells by centrifugation, they were then shifted to a new medium containing 100–200 mM CuSO_4_ (Cu from now on) or 1 mM CdSO_4_ (Cd from now on) during 24 h. After this time, cells were treated to obtain protein extracts. Growth was monitored by measuring unstained cells numbers by means of a Petroff-Hausser chamber under a phase contrast microscope.

### 2.2. Preparation of Protein Extracts from *S. metallicus*

Cells from 800 mL of a control culture grown to 10^8^ cells/mL (early stationary phase), or cultures shifted to 100–200 mM Cu or 1 mM, were harvested by centrifugation at 7,700 g for 15 min. The pellets were washed with medium 88 to remove the sulfur. Cells were then resuspended in 50 mM Tris-HCl pH 8.15, 10 mM EDTA, 100 *μ*g/mL PMSF buffer (20 *μ*L per mg wet weight), frozen, and sonicated six times for 30 s each time. The lysates were centrifuged at 4,300 g for 5 min to eliminate cellular debris. The protein concentration of supernatants was determined by the method of Bradford (Coomasie Plus protein assay reagent, Pierce). Between 120 and 500 *μ*g of proteins from the protein extracts were mixed with rehydration IEF buffer, as described by Hatzimanikatis et al. [31] and Choe and Lee [32] with some modifications, including urea 8 M, thiourea 2 M, CHAPS 2% (w/v), Bio-Lyte 3–10 0.27% (v/v), Bio-Lyte 5–8 0.13% (v/v), and bromophenol blue 0.001% (w/v), followed by incubation at 25°C for 30 min. DTT (0.03 g) and sterile nanopure water were then added to complete a final volume of 300 *μ*L. The samples were then incubated at room temperature for 1 h.

### 2.3. Isoelectric Focussing (IEF)

Each sample (300 *μ*L) was loaded onto the pH 3–10 (nonlinear) 17 cm IPG strips (Biorad) in the first dimension chamber and was incubated at room temperature for 1 h. Mineral oil (2.5 mL) was then added to prevent evaporation of the sample and precipitation of urea, and strips were passively rehydrated for 18 h. The isoelectric focusing was performed with the PROTEAN IEF (Biorad) using the following conditions: 250 V for 15 min, 2,000 V for 2 h, 8,000 V for 4 h, 10,000 V for 11 h, and 50 V for 4 h, reaching a total of 120,000 V/h.

### 2.4. SDS-PAGE

Prior to the second-dimensional electrophoresis, strips were equilibrated as described by Hatzimanikatis et al. [31] with some modifications. Strips were incubated for 15 min with a solution containing 6 M urea, 156 mM DTT, 30% (v/v) glycerol, 2% (w/v) SDS and 24 mM Tris-HCl pH 6.8 and subsequently for 15 more min in a solution containing 6 M urea, 135 mM iodoacetamide, 30% (v/v) glycerol, 2% (p/v) SDS, and 24 mM Tris-HCl pH 6.8. Finally, strips were incubated with electrophoresis buffer containing 192 mM glycine, 1% (w/v) SDS, and 250 mM Tris-HCl pH 8.3 until the second-dimensional run. SDS-PAGE was performed using the PROTEAN II xi cell as described by Laemmli [33], and gels consisted of 11.5 or 15% (w/v) polyacrylamide. Strips were then overlaid onto the second-dimensional gels sealed with 1% (w/v) agarose in electrophoresis buffer containing a trace amount of bromophenol blue. Electrophoresis was carried out at constant 70 V for 15 h. All experiments were performed in triplicate. Gels were stained with silver or Coomasie Blue G-250 as described by Shevchenko et al. [34] and Giavalisco et al. [35], respectively. 

### 2.5. Gels Analysis and Mass Spectrometry

Gel images were digitized by scanning (Epson) and analyzed with the Delta 2D software (Decodon) to identify the spots differentially expressed due to the presence of toxic metals. An estimate of relative quantitative changes was made on the basis of the change in percent volume among silver stained gels. Spots of interest were recovered from Coomasie Blue G-250 stained gels manually and were sent to electron spray tandem ionization mass spectrometric analysis (tandem MS-MS: ESI-QUAD-TOF). The results obtained were analyzed with Mascot algorithm (http://www.matrixscience.com/index.html) by using the MS/MS Ion search, and all genomes available at databases were used as queries. The entire MS analysis was performed at the Cambridge Center for Proteomics, University of Cambridge, UK.

### 2.6. CODEHOP-PCR

CopA and CopM amino acid sequences from *S. acidocaldarius*, S*. tokodaii* and *S. solfataricus* were obtained from NCBI (http://www.ncbi.nih.gov/). After aligning the sequences by using the CLUSTAL X program to identify areas of homology, consensus-degenerate PCR primers were designed according to the CODEHOP strategy [36, 37], using the WWW access at http://blocks.fhcrc.org/blocks/codehop.html. Two regions of high sequence similarity were identified for both CopA and CopM sequences, respectively (Figure S1 see Supplementry Material 

available online at http://dx.doi.org/10.1155/2013/289236) and used to design the consensus-degenerate hybrid oligonucleotide primers (Table 1). Consensus-degenerate hybrid oligonucleotide primers were designed for the N- and C-terminus of CopA, while a pair of degenerate hybrid oligonucleotide primers was designed for CopM (Figure S1).

Amplification reactions contained 1x thermophilic DNA polymerase buffer with 2 mM MgCl_2_, 0.2 mM dNTPs, 0.5 *μ*M of each primer, 5 U Taq DNA polymerase, 40 ng of *S. metallicus* genomic DNA as a template and water to a final volume of 50 *μ*L. The thermal cycling conditions were 3 min at 95°C; following 30 cycles of 95°C for 30 s, 50°C for 30 s, and 72°C for 1 min; 1 final additional cycle at 72°C for 10 min. Products of amplification were applied onto 1.0% (w/v) agarose gels, and main amplification bands were excised, purified, and TA-cloned into pCR2.1-TOPO (Invitrogen) vector and finally sequenced.

### 2.7. Genome Walking Experiments

Genome walking strategy was performed as described by Acevedo et al. [38]. Thus, a double-stranded oligo-cassette AdaptT adapter was constructed by annealing of the two unphosphorylated primers AdaptF: (5′-CTAGGCCACGCGTCGACTAGTACTA-GCTT-3′) and AdaptR: (5′-AGCTAGTACTAGTCGACGCG-TGGCCTAG-3′). Annealing was performed by heating the primers (10 *μ*M) in a boiling water bath for 5 min, and then slowly cooling to room temperature.

Six different DNA libraries were constructed by means of digesting 1 *μ*g of *S. metallicus* genomic DNA with the following restriction enzymes: *Hind*III, *Bam*HI, *Eco*RI, *Eco*RV, *Nco*I, and *Pst*I, respectively. DNA digestion reactions were carried out using 10 U of restriction enzyme, 2 *μ*L of the corresponding enzyme reaction buffer in 20 *μ*L of total reaction volume. The reaction mix was incubated at 16°C during 16 h. To complete the 3′ recessive end of the DNA fragments and to add a 3′ overhanging adenine, 500 ng of the digested and purified DNA were incubated with 5 U of Taq DNA polymerase, 1 *μ*L of 10 mM dNTPs mix, and 5 *μ*L of 10x thermophilic DNA polymerase buffer in 50 *μ*L total volume, at 70°C for 45 min. Seven *μ*L of this mixture was then incubated with 15 pmol of AdaptT oligo-cassette, 1 U T4 DNA ligase (Promega) and 2 *μ*L of 5x ligase buffer, in a total volume of 10 *μ*L. The ligation reaction was incubated at 16°C during 16 h.

Two consecutive amplification reactions were then performed. The first PCR reaction was done with 1x Elongase mix buffer, 1.9 mM MgCl_2_, 0.2 mM dNTPs, 0.5 *μ*M first specific primer (SP1) (designed from the known sequence of the target gene; a forward primer was used to amplify the 3′ end a reverse primer for 5′ end amplification), 5 *μ*L of the ligated DNA diluted 10-fold and 1 *μ*L of Elongase (Invitrogen) and water to a final volume of 50 *μ*L. The thermal cycling conditions were as follows: 1 cycle at 94°C for 1 min, 20 cycles of 94°C for 32 s and 68°C for 5 min, and one final additional cycle at 70°C for 7 min. The PCR product was diluted 10 fold and 3 *μ*L were used as a DNA template for a second PCR, which was performed using the same conditions as the first PCR with 0.5 *μ*M second specific primer (SP2) and 0.2 *μ*M oligo-cassette-specific primer AdaptF2 (5′-CACGCGTCGACTAGTACTAGCTT-3′) and 1 *μ*L of Elongase. The thermal cycling conditions were 1 cycle at 94°C for 1 min, 35 cycles of 94°C for 32 s, and 68°C for 5 min, and 1 final additional cycle at 70°C for 7 min. PCR products were excised, purified, TA-cloned into pCR2.1-TOPO (Invitrogen) vector, and finally sequenced.

### 2.8. Isolation of Total RNA

Cell pellets (10 mg wet weight) were collected and diluted in 60 *μ*L TEN buffer (20 mM Tris-HCl pH 8.0, 1 mM EDTA, 100 mM NaCl) followed by addition of 60 *μ*L TENST buffer (20 mM Tris-HCl pH 8.0, 1 mM EDTA, 100 mM NaCl, 1.6% Na-lauroyl sarcosine, and 0.12% Triton X-100). This suspension was incubated at room temperature for 15 min to allow cell lysis. Total RNA was then extracted using the TRIzol reagent (Invitrogen) as recommended by the manufacturer. DNA contamination in RNA preparations was removed by DNase I treatment (Roche), following the manufacturer's instructions. RNA was then purified, precipitated, and finally resuspended in diethylpyrocarbonate (DEPC)-treated water. Total RNA concentrations were estimated by spectrophotometric measurements (OD_260_) and its quality was evaluated by determining the ratio of absorption at 260 nm and 280 nm, which was within the preferred range of 1.8–2.1.

### 2.9. Northern Blotting

For differential gene expression by Northern blotting experiments, the media were supplemented with either different concentrations of Cu (5–50 mM), Cd (5 mM), NiSO_4_ (15 mM), ZnSO_4_ (50 mM), or Ag_2_SO_4_ (0.08 mM), respectively, as indicated. For some experiments, elemental sulfur was replaced by a chalcopyrite (CuFeS_2_) concentrate, which was used as the only energy source at 1% (w/v). 

Total RNA (5 *μ*g) was separated by electrophoresis on a 1% formaldehyde agarose gel followed by blotting onto Hybond-N nylon membranes (Amersham Pharmacia Biotech, Bucking-hamshire, UK). Hybridization was conducted as described by Sambrook et al. [40]. DNA probes were labeled by random primer DNA labeling kit (Fermentas) using [*α*-32 p] dCTP. The hybridization signal was detected and analyzed by using the molecular Imager FX system and Quantity One software. The primer sequences for amplification of these gene probes are listed in Table 1.

### 2.10. Cotranscriptional Analysis by RT-PCR

To study the expression of adjacent genes *copM* and *copA,* cDNA was synthesized by using 0.8 *μ*g of total RNA from a *S. metallicus* culture grown in the presence of 20 mM Cu and a reverse primer hybridizing to *copA* sequence. A forward primer annealing on *copM* sequence was used for the PCR reactions (Table 1). PCR amplifications were performed with 1 *μ*L of a 1/10 dilution of the cDNA and 25 pmol of each primer. Amplification conditions included an initial 3 min of denaturation at 95°C, followed by 35 cycles of 30 s at 95°C, 30 s at 55°C, and 1.5 min at 72°C and finished by 10 min at 72°C. A reverse transcriptase reaction without enzyme was carried out in order to exclude amplification due to genomic DNA contamination.

### 2.11. Quantitative RT-PCR

Single stranded cDNA was synthesized from 0.8 ng of DNA-free RNA samples using random hexamers (Fermentas) and ImProm-II reverse transcription system (Promega) following manufacturer's instructions. The software tool IDT Scitools (Integrated DNA Technologies) was used to design primers producing amplicons of 150–200 bp (Table 1). qPCR was performed with 2 *μ*L of 1 : 10 diluted cDNA samples, 12.5 *μ*L 2x QuantiFast SYBRGreen PCR Master Mix (Quiagen), 1 *μ*M of each primer, and water to a final volume of 25 *μ*L. The efficiency of each primer pair was calculated from the average slope of a linear regression curve, which resulted from qPCRs using a 10-fold dilution series (10 pg–10 ng) of *S. metallicus* chromosomal DNA as template. Cq values (quantification cycle) were automatically determined by Real-Time Rotor-gene 6000 PCR software (Corbett Life Sciences) after 40 cycles. Cq values of each transcript of interest was standardized to the Cq value of the 16S *rRNA* gene [39]. At least 3 biological replicates of each assessed condition and 2 technical replicates per qPCR reaction were performed. 

## 3. Results and Discussion

### 3.1. Tolerance of *S. metallicus* and *S. solfataricus* to Cu and Cd

**Figure 1:**
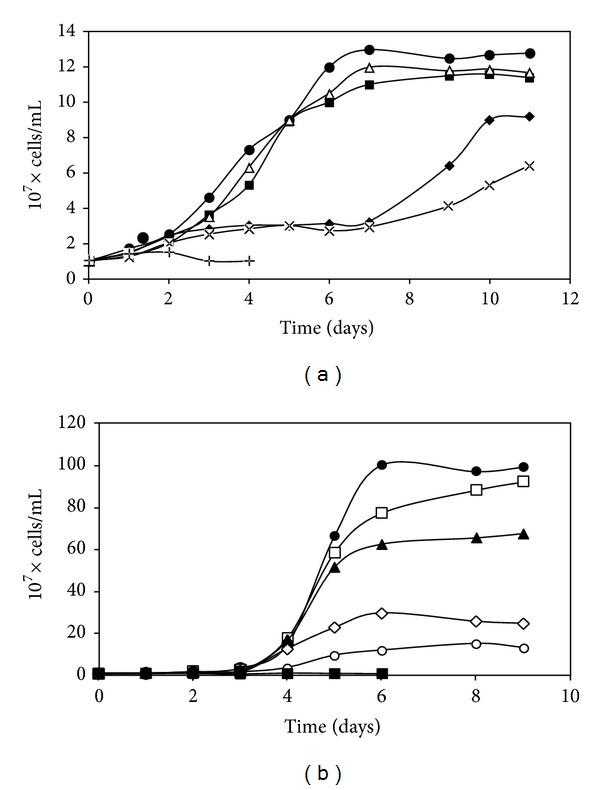
Growth of *S. metallicus* (a) and *S. solfataricus* (b) in the presence of Cd. Cells were inoculated in their respective growth media in absence of added Cd (●), or were supplemented with 0.005 mM (□), 0.01 mM (▲), 0.05 mM (*◊*), 0.1 mM (*⚪*), 0.5 mM (■), 1 mM (△), 2 mM (♦), 3 mM (×), or 5 mM (+) Cd, and cells were counted daily.

In a previous work, it was determined that *S. metallicus* was able to tolerate high Cu concentrations. While the presence of 100 mM Cu did not affect *S. metallicus* growth kinetics, a decreased in cell biomass of only 30% was observed when exposed to 200 mM Cu [30]. The high tolerance to Cu has been described in other acidophilic Bacteria and Archaea compared mostly with neutrophilic microorganisms [11, 14]. Here, this analysis was further extended to characterize the response of *S. metallicus* to Cd. Thus, it was determined that *S. metallicus* growth was not affected in the presence of either 0.5 or 1 mM Cd when compared with the control condition in the absence of the metal (Figure 1(a)). In addition, when *S. metallicus* was challenged with 2 and 3 mM Cd, it was observed that at the late exponential growth phase, the cell numbers decreased by 30 and 50%, respectively (Figure 1(a)). Moreover, growth of *S. metallicus* was completely inhibited in the presence of 5 mM Cd (Figure 1(a)). On the other hand, *S. solfataricus* was not able to grow at Cd concentrations greater than 0.05 mM (Figure 1(b)). At 0.01 mM Cd, *S. solfataricus* cell numbers decreased around 35% (Figure 1(b)). These results are in agreement with previous reports in which *S. solfataricus* was shown to be able to grow in up to 0.01 mM Cd [41]. Other acidophilic archaeons involved in bioleaching processes, such as *Metallosphaera sedula* and *Ferroplasma acidarmanus*, were found to be able to tolerate up to 0.9 and 9 mM Cd, respectively [18, 42]. The minimal inhibitory concentration (MIC) to Cd has been described to be not higher than 1 mM for several thermophilic and neutrophilic *Thermococcus* species [43]. Recently, it was reported that the most radioresistant archaeon, *Thermococcus gammatolerans*, stands Cd concentrations with an MIC of 2 mM [27]. To date, despite the heavy metals tolerance showed by some of these microorganisms, strategies to withstand stress from transition metals have been most widely studied only in haloarchaea [44].

### 3.2. Effect of Cu and Cd on the Global Proteome of *S. metallicus*

**Figure 2:**
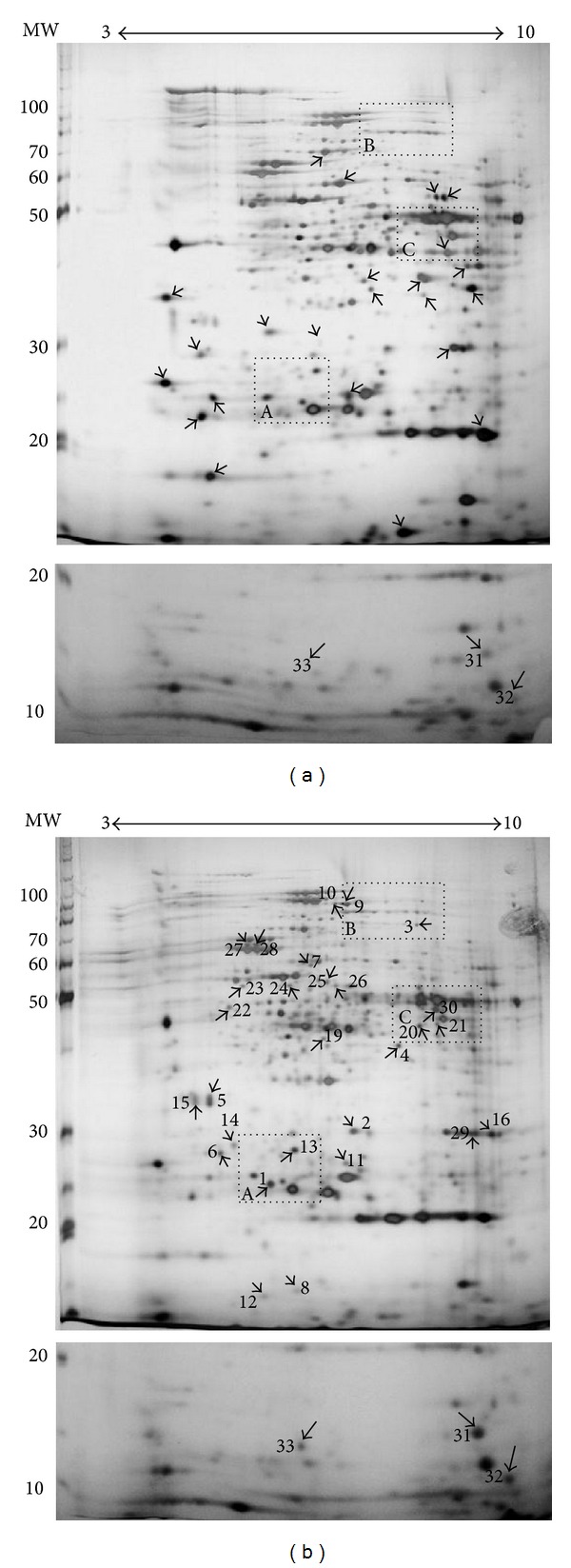
Changes in the proteome of *S. metallicus* grown in the presence of Cu. Cells were incubated in the absence of any added metal (a) or in the presence of 100 mM Cu (b). Arrows indicate the spots that were downregulated (a) or upregulated (b) in the presence of Cu. Numbers indicate the spots with increased intensity in cells treated with copper. The dashed boxes are shown as enlarged areas that include some proteins upregulated in the presence of Cu and Cd (Figure 3). The bottom panels show low molecular weight proteins upregulated in the presence of Cu separated by using a 15% polyacrylamide gel in the second dimension.

**Figure 3:**
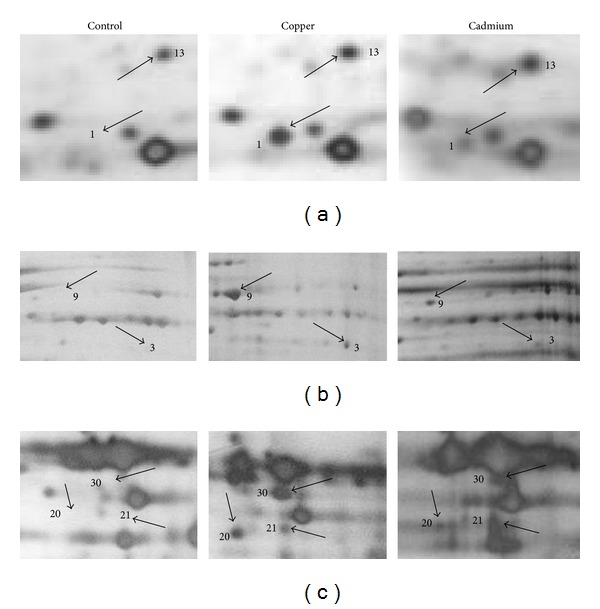
Comparison of selected proteins of *S. metallicus* cells treated with Cu or Cd. Protein extracts were obtained from cells treated without metals and with 100 mM Cu or 1 mM Cd for 24 h and the proteins were separated by 2D-PAGE. The segments (a), (b), and (c) are the enlarged dashed boxes in Figure 2 under the three conditions indicated. Numbers show some of the spots with increased intensity in cells treated with Cu or Cd.

The proteomic response to Cu and Cd was analyzed to identify proteins which could be involved in heavy metals resistance in *S. metallicus*. Early stationary phase growing cells were untreated or treated with either 100 or 200 mM Cu or 1 mM Cd during 24 h, and analyzed by comparative two-dimensional gel electrophoresis as indicated in experimental procedures. Twenty-three proteins were found to be down-regulated after 100 mM Cu treatment (Figure 2(a)). A similar pattern was obtained at 200 mM Cu (not shown). Furthermore, by using the Delta 2D software (Decodon), eleven of these proteins were found to be completely absent in the gels and more than 8 proteins decreased their intensity in the range from 1.5-to 5-fold compared with control cells grown in the absence of Cu (Figure 2(a)). Therefore, these results show that a number of proteins became non-detectable or decreased their levels when *S. metallicus* faced Cu. This kind of response was also seen when the microorganism was exposed to Cd (data not shown). This behavior has also been observed in similar studies where *F. acidarmanus* was challenged by either As(III) or Cu [16, 18]. In addition, the expression of 30 other proteins was found to be upregulated after Cu treatment (100 or 200 mM) (Figure 2(b)). Most of these proteins could be identified only in the condition with Cu, and spots 5, 13, and 15 were induced 2.6, 3.4, and 5.5-fold, respectively, compared to cells without Cu treatment. Moreover, 3 proteins of low molecular weight (spots 31, 3, and 33) that increased their expression when cells were exposed to 100 mM Cu were also identified (Figure 2, bottom panels). These proteins were only detected when 15% polyacrylamide was used during the second-dimensional separation (SDS-PAGE). On the other hand, a large decrease in overall expression of proteins after 1 mM Cd treatment was observed (not shown). Interestingly, 10 out of the 13 proteins identified as up-regulated when cells were exposed to Cd and also showed increased levels after Cu treatment. Some of them are shown in Figure 3.

### 3.3. Identification of Proteins Upregulated in Cells Treated with Cu and Cd in *S. metallicus*

A total of 18 *S. metallicus* proteins whose levels were found to be up-regulated in response to Cu were analyzed by mass spectrometry. The identified proteins included functions related to the production and transport of energy, biosynthesis of amino acids, stress responses, and transcription regulation (Table 2). Amongst the proteins related to production and transport of energy, a putative ATP synthase subunit B (spot 23) that has been described as playing a fundamental role in ATP synthesis was identified (Table 2). When cells are subjected to some stressing conditions such as the presence of heavy metals, a greater cellular demand for energy has been reported to occur [18]. Most likely, this phenomenon might be due to ATP-driven Cu transport via ATPases that have a substantial interplay during metal detoxification [45]. Furthermore, the ATP synthase subunit B was also identified as up-regulated when *S. metallicus* was treated with Cd (Table 2). 

Two other proteins corresponded to putative oxidoreductases such as ferredoxin oxidoreductase (spot 3) and alcohol dehydrogenase (spot 4), that have been generally involved in electron transporter chains and use NAD^+^ as an electron acceptor. The levels of ferredoxin oxidoreductase were also up-regulated in response to Cd (Table 2). Several previous reports have suggested that oxidoreductases contribute to an oxidative protection in both Bacteria and Archaea in response to heavy metals [46–48]. In the neutrophilic microorganisms *Escherichia coli* and *Staphylococcus aureus*, it has been described that oxidases and dehydrogenases contribute to oxidative protection, Cu homeostasis, and stress responses [46, 47]. Furthermore, some proteins involved in oxidative damage repair, such as NADH-dependent oxidases and thioredoxin reductases, were expressed in cells of *F. acidarmanus* exposed to As(III) [16]. The expression of this group of proteins has also been observed when the same microorganism was exposed to Cu [18]. Therefore, oxidoreductases play an important role against oxidative stress and may eliminate reactive oxygen species, which constitute the major component of the stress caused by transition metals [44, 49, 50].

The enzymes related to biosynthesis of amino acids were found as commonly up-regulated either in response to Cu or Cd. They corresponded to a phosphoglycerate dehydrogenase (spot 9) and a glutamate dehydrogenase (spot 20). The former one catalyzes the NAD^+^-dependent oxidation of 3-phosphoglycerate into 3-phosphohydroxypyruvate, a branch point from the glycolytic pathway and the initial reaction in L-serine biosynthesis [51]. Glutamate dehydrogenase catalyzes the oxidative deamination of glutamate to produce 2-oxoglutarate and ammonia with reduction of NAD^+^ [52]. Proteins involved in amino acids synthesis were expressed in cells of *F. acidarmanus* exposed to As(III) [16]. Moreover, increased levels of proteins involved in the biosynthesis of sulfur-containing amino acids have been observed in *Saccharomyces cerevisiae* cells exposed to Cd [24], suggesting that these proteins might also have a role in the cellular response to adverse conditions.

Two proteins showed identity to components of the stress response mechanism such as one subunit of the HSP60 chaperonin (spot 24) and one subunit of the proteasome (spot 1). In Archaea, the stress protein HSP60 is directly involved in protein folding processes [53]. Proteomic analysis of *F. acidarmanus* cells exposed to As(III) and Cd revealed the expression of proteins involved in protein folding and DNA repair, including HSP60 chaperonin and DnaK heat-shock protein (HSP70); thereby the authors suggested that this microorganism uses multiple mechanisms to resist high levels of Cu [16, 18]. On the other hand, proteasomes are described as nonspecific proteolytic nanomachines associated with protein catabolism. Proteasomes are known to be closely involved in maintaining protein quality control by degrading miss folded and denatured proteins in response to cell stress in all three domains of life [54]. A proteomic analysis in the thermoacidophilic archaeon *Thermoplasma acidophilum* showed that proteasomes and chaperone-related proteins were highly induced against stress conditions, indicating a high turnover rate of proteins [55]. In *P. furiosus*, the expression of the *β*1 subunit of the proteasome was induced in cells subjected to heat shock stress [56]. Additionally, the proteasome subunit was also found to be up-regulated when *S. metallicus* was subjected to Cd stress (Table 2). 

The transcriptional regulation-related proteins corresponded to a putative transcription regulator (spot 32) and the transcription factor NusA (spot 33) involved in the intrinsic termination of transcription [57]. NusA has been also reported to display important functions in the cellular response to adverse factors [58]. The expression of two transcriptional regulators related to amino acids biosynthesis and metal-dependent genetic repression were found to be induced in *P. furiosus* cells subjected to oxidative stress [48]. Moreover, the expression of several genes encoding predicted transcriptional regulators were induced following Cd exposure in *T. gammatolerans *cells [27].

Spots 5, 6, 13, 14, 15, 21, 22, 30, and 31 (Table 2) did not show significant scores with any protein sequences currently available in the data bases. Interestingly, spots 2, 4, 9, 13, 21, and 30 were found to be commonly upregulated when *S. metallicus* cells were treated with either Cu or Cd (Figure 3, Table 2).

The results presented here are consistent with previous work describing global protein expression profiles in response to heavy metals as reported for *F. acidarmanus* [16, 18], microbial-biofilm communities in an acid mine drainage site [59], and other prokaryotic and eukaryotic microorganisms [24, 60]. Therefore, a coordinated expression of different groups of genes suggests the existence of regulatory networks such as stress response mechanisms and respiratory chain adjustments to cope with the presence of heavy metal ions [61]. 

### 3.4. Cu-Resistance (Cop) Genes Cluster Is Duplicated in *S. metallicus* Genome

**Figure 4:**
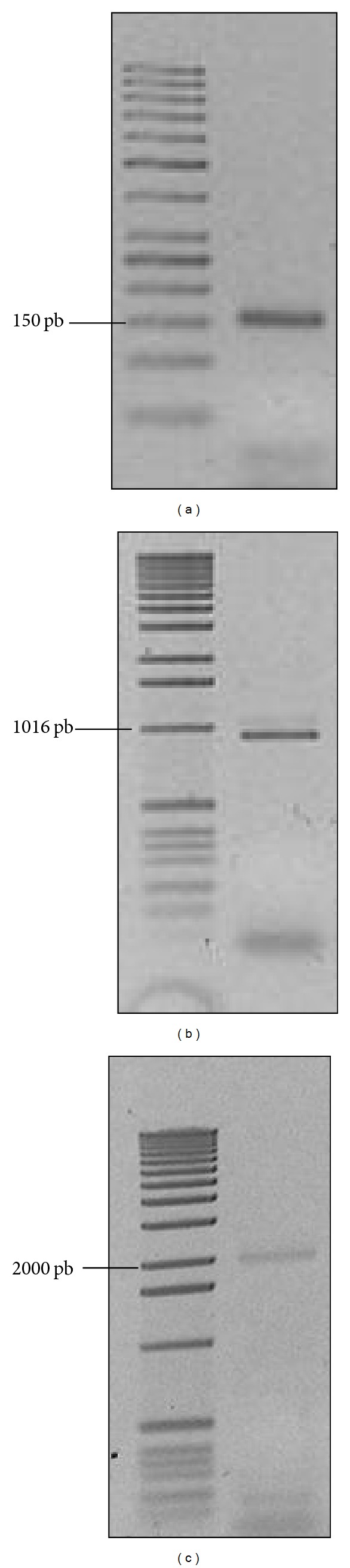
CODEHOP-based PCR for the amplification of the putative genes* copA* and *copM* from *S. metallicus*. Amplification products, (a) using degenerate PCR primers (DOP) copM_degF and copM_degR designed from amino acid sequence conserved blocks of *Sulfolobales* CopM proteins, (b) using primers copA_cdegF and copA_cdegR designed by CODEHOP strategy for amplification of a *copA*-like gene. (c) Primers copM_degF and copA_cdegR were assesed for the amplification of a *copMA*-like DNA region. Those primers were tested with genomic *S. metallicus* DNA samples. Amplicons expected sizes were as follows: ca. 150 bp for *copM, *ca. 950 bp for *copA*, and ca. 2,000 bp for *copMA*. PCR products were excised and cloned for later sequencing.

A Cu-resistance (*cop*) locus has been described to be highly conserved in archaeal genomes, which consists of genes encoding a new type of archaeal transcriptional regulator (CopT), a metal-binding chaperone (CopM), and a Cu-transporting P-type ATPase (CopA) [19]. Since the genome sequence of *S. metallicus* is not yet available, we searched for the presence of *cop *genes by using consensus degenerate hybrid oligonucleotide primers-based PCR (CODEHOP-PCR). CopA and CopM amino acid sequence alignments from *S. acidocaldarius*, *S. tokodaii, *and* S. solfataricus *were used to identify conserved sequence blocks and therefore to design either CODEHOP or DOP (degenerate hybrid oligonucleotide) primers (Figure S1,Table 1). Thereby PCR assays using *S. metallicus* genomic DNA as template yielded amplicons of ca. 150 bp and ca. 950 bp for *copM* and *copA* CODEHOP pair primers, respectively (Figures 4(a), 4(b)). As estimated from primer pair's position on amino acid sequence alignments, the obtained PCR products corresponded to the expected sizes, suggesting the presence of *copM-* and *copA*-like genes in *S. metallicus* genome. In order to determine the identity of the amplified DNA fragments, they were TA-cloned and sequenced. Blastx sequence analysis showed that the 150 bp DNA fragment coded for an incomplete ORF sharing 63% homology with *S. solfataricus* CopM (SSO10823), whereas the analysis of the 950 pb DNA fragment yielded a partial ORF sharing 54% homology with *S. solfataricus* CopA protein (SSO2651). 

The genomic organization of the putative *cop* genes in *S. metallicus* was analyzed to find out its similarity with those previously described in *S. solfataricus* P2 and *F. acidarmanus*, where the *cop* cluster consists of tandem-orientated genes as *copTMA *[18, 19]. Thus, using copM_degF (forward) and copA_cdegR (reverse) primers, we attempted to amplify a putative *copMA* DNA region. This PCR yielded a product of ca. 2,000 pb, corresponding to the expected size, and resulted in 2 ORFs showing high sequence homology with CopM and CopA from *S. solfataricus* (Figure 4(c)). Interestingly, when aligning this *copMA* sequence with the *copA* and *copM* sequences obtained previously, they were not identical, which strongly indicated the presence of duplicated putative *cop* genes encoding for both Cu-ATPases (*copA1* and *copA2*) and metallochaperones (*copM1* and *copM2*) in the *S. metallicus* genome. 

To isolate the entire *cop* gene sequences, the genome walking method described by Acevedo et al. [38] was used. Six different DNA libraries were constructed from *S. metallicus* genomic DNA digested with several restriction enzymes, including 5′ overhang and blunt end restriction enzymes. The DNA libraries were used as templates in 2 successive PCR reactions. From the previously known sequences, two specific sense primers for the 3′ end amplification and two specific antisense primers for the 5′ end amplification were designed (abbreviated SP1 and SP2). Therefore, the 5′ and 3′ ends of the putative genes *copA1* and *copA2* that had been partially identified through the genome walking technique were amplified.

**Figure 5:**
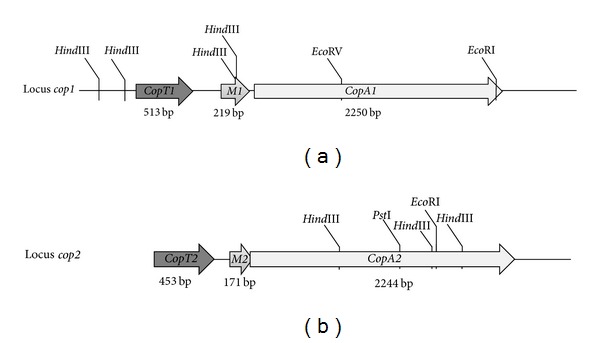
Schematic representation of the two *cop* gene clusters isolated from *S. metallicus* genome. Each cluster codes for an *Archaea*-specific transcriptional regulator (*copT*), a metallochaperone (*copM*), and a P-type Cu-exporting ATPase *(copA*). Lengths of each gene are indicated. Locus *cop1* corresponds to 4,346 sequenced base pairs and shows an intergenic region *copT1-M1* of 232 base pairs. Locus *cop2* corresponds to 3,435 base pairs and shows 89 base pairs *copT2-M2* intergenic region. Genes *copM2* and *copA2* overlapped in 11 base pairs. *copT2* was not fully isolated.

By overlapping the sequence isolated by degenerate PCR and the lateral sequences obtained by genome walking (Figure S2), it was possible to complete the whole nucleotide sequence of the putative gene *copA1*. Additionally, it was possible to confirm the presence of the upstream *copM1* gene and determine the occurrence of a partial *copT1* gene tandem-orientated upstream of *copM1* (Figure 5). Through additional genome walking experiments, the partial *copT1* sequence could be further completed. The same was true when isolating the whole nucleotide sequence of the putative gene *copA2 *as the presence of *copM2 and copT2 *genes were confirmed upstream of *copA2. *However, it was not possible to complete the whole 5′ end of the putative gene *copT2 *as its determined length corresponded to only 90% of the full length when compared with *S. solfataricus copT* gene (Figure 5). 

In conclusion, degenerate PCR together with genome walking experiments allowed to describe the occurrence in *S. metallicus* genome of 2 *cop* genes loci (named as locus *cop1* and locus *cop2*) coding for paralogous genes whose products may be involved in Cu-resistance (Figure 5). Although some archaeal genomes exhibit two copies of putative Cu-P-type ATPases as described for *S. solfataricus* [62], the genomic arrangement displayed as a *cop* gene cluster (*copTMA*) has been reported to be represented in only one copy for many archaeal genomes [10, 19, 21]. Moreover, we further updated this analysis by searching for duplications of the *cop* genes cluster in all available archaeal genome sequences up to date (October, 2012), retrieving only one *cop* locus in each case. Thereby we propose that the discovery of this *cop* gene cluster duplication in the genome of *S. metallicus* constitutes so far an unprecedented feature for a representative of the *Archaea* domain and might contribute to its high metal resistance. 

In this context, it has been widely reported that increased gene copy number can increase gene expression allowing prokaryotic to thrive under growth limiting conditions [13, 63–68]. However, alongside the augmented gene copy, one cannot exclude that the *cop* operon duplication in *S. metallicus* may offer among others: a wider repertoire of Cu-ATPases, which might differ in terms of metal affinities and/or specificity, efflux rates, and/or differences in their abundance regulation. 

Throughout amino acid sequence analysis, it was further determined that each polypeptide encoded by *S. metallicus*, with the exception of CopT1, showed to be highly homologous to their orthologous counterparts encoded by others *Sulfolobales* (Table 3). Moreover, the 3 ORFs encoded by the locus *cop2* shared about 90% identity with the corresponding *S. solfataricus* orthologous peptides (Table 3). *S. metallicus cop* genes products showed characteristic domains, referred to as critical for their respective proposed biological activities. *S. metallicus* paralogous gene products CopA1 (749 aa′) and CopA2 (747 aa′) shared 51.3% of identity. These two putative Cu-ATPases (CopA1 and CopA2) contain the amino acid sequence motif CPCALGLA which has been proposed to confer Cu-transporting specificity in CPx-ATPases [48] and has been also found in other CopA-like proteins from biomining Bacteria and Archaea [14].

On the other hand, CopM1 (71 aa) and CopM2 (56 aa), sharing 66% of identity, present the proposed metal binding domain TRASH (trafficking, resistance, and sensing of heavy metals) [10]. Additionally, when analyzing the putative transcriptional regulators, the presence of a C-terminal TRASH domain in both CopT1 and CopT2 was also found. CopT1 sequence also contains an entire N-terminal helix-turn-helix (HTH) motif (not shown) that resembles the DNA-binding motifs of prokaryotic transcriptional regulators, such as Lrp-like proteins [67]. The HTH motif was only partially identified in CopT2 since the 5′ region of *copT1* gene has not been yet fully isolated. 

### 3.5. Transcriptional Analysis of *S. metallicus* Cop Genes

**Figure 6:**
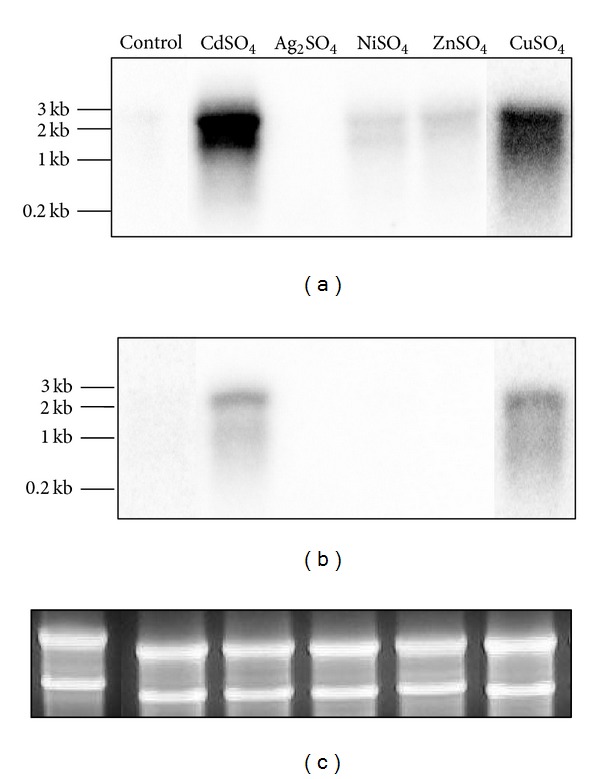
Northern blot analysis to determine the expression of *S. metallicus copA1* (a) and *copA2 *(b) putative genes in response to various metals. *S. metallicus *total RNA was extracted from cultures grown either in the absence (control) or the presence of CuSO_4_ (50 mM), Ag_2_SO_4_ (0.08 mM), NiSO_4_ (15 mM), ZnSO_4_ (50 mM), and CdSO_4_ (2 mM). P^32^-radioactivelly labelled DNA fragments annealing to *copA1* and *copA2 *sequences, respectively, were used as probes. (c) shows rRNAs as total RNA loading control.

To get insight into the role of the two *cop* loci, the expression of the corresponding genes was analyzed under various conditions. *S. metallicus* was first grown in presence of different heavy metals (Cu, Zn, Cd, Ni o Ag) at a given concentration that did not affect growth kinetics [30, 42]. Subsequently, total RNA was isolated from late exponentially grown cultures and Northern blot experiments were carried out in order to determine the expression of both *copA1* and *copA2 *genes. As depicted in Figure 6, transcription of both *copA1* and *copA2 *messengers were specifically induced in the presence of Cu and Cd ions. This gene expression pattern is in good agreement with what has been described for *S. solfataricus*, where the bicistronic *copMA* transcript levels were also found to be up-regulated in response to both Cu and Cd [19].

**Figure 7:**
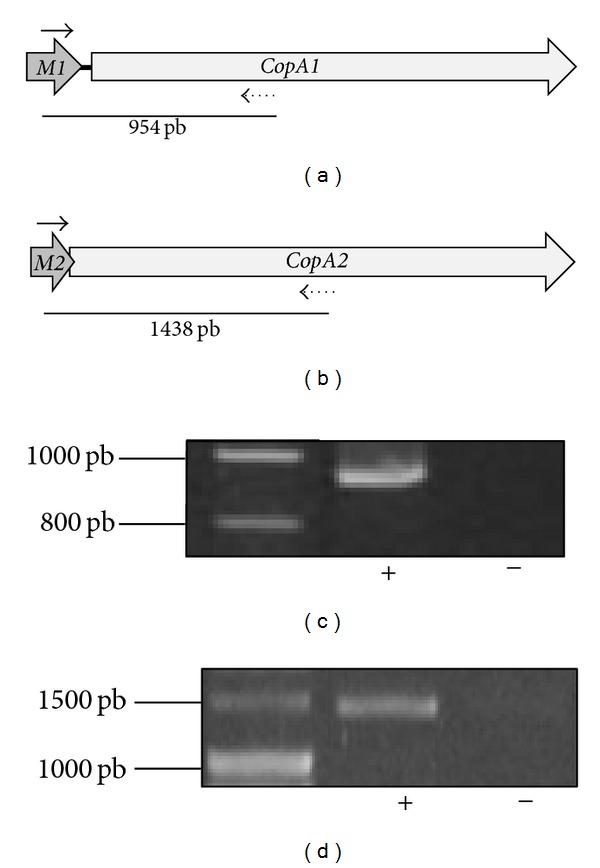
Cotranscription analysis of *copMA1* and *copMA2* genes. cDNA was synthesized with a reverse primer (dotted arrows) hybridizing toward the 3′ end of either *copA1* (a) or *copA2* (b).* S. metallicus* total RNA was extracted at the late exponential phase from a culture growing in presence of 20 mM Cu. PCR amplifications were carried out with these cDNAs and each corresponding primer pair (black arrows) as listed in Table 1. (c) and (d) show RT-PCR products obtained for the *copMA1* and *copMA2 *intergenic regions, respectively. Reverse transcriptase reactions with (+) and without (−) the Improm II reverse transcriptase enzyme were carried out in order to exclude amplification due to genomic DNA contamination. Expected sizes (in base pairs) for the corresponding PCR products are given in (a) and (b).

**Figure 8:**
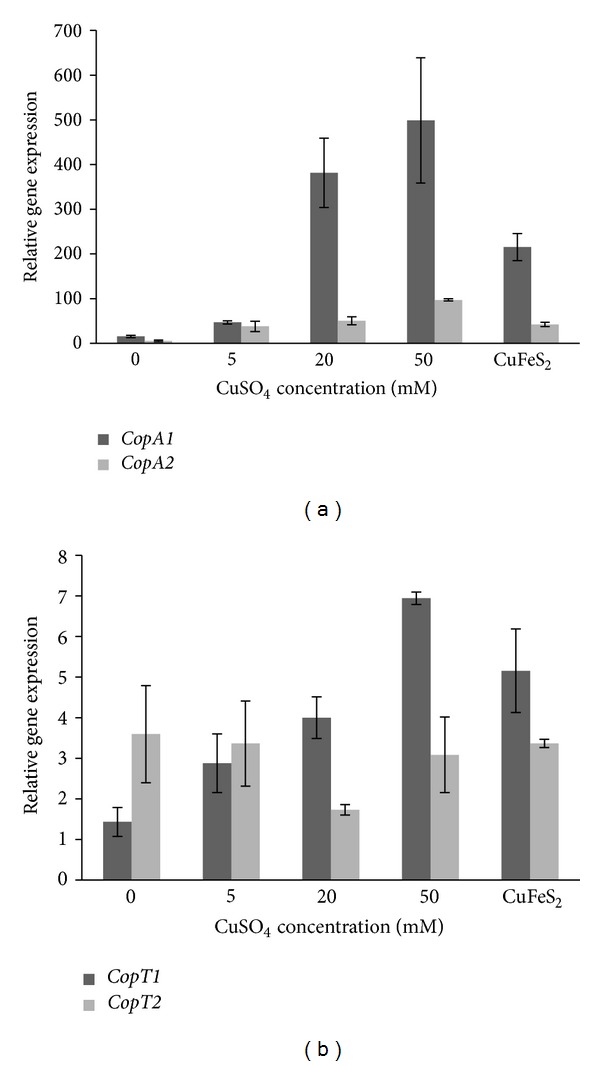
Relative expression levels of *S. metallicus *genes* copA1 *and *copA2 *(a) *copT1* and *copT2 *(b). Expression of *S. metallicus cop *genes wasassessed by qRT-PCR and normalized against 16S rRNA gene expression in individual cultures. Mean values and standard deviations are from analyses of three independent cultures grown in the presence of 0, 5, 20, 50 mM Cu or in 1% of a chalcopyrite ore (CuFeS_2_) concentrate.

Northern blot analysis showed that the sizes seen for both *copA* transcripts (~2.5 Kb) did not correspond exactly with the individual genes length (each of about 2.25 Kb) (Figure 6). This strongly suggested that the transcripts could also include the respective *copM* gene located upstream of *copA* in each *cop *loci. To confirm this assumption, a co-transcription experiment was done (Figure 7) in which the cDNAs were obtained by using RNA extracted from a culture grown in the presence of 20 mM Cu and using a reverse primer hybridizing with *copA1* (Figure 7(a)) or *copA2* (Figure 7(b)) gene sequence, respectively. PCR amplifications were carried out by using the corresponding cDNAs as templates and each pair of primers lying in adjacent genes (*copM*). The presence of an amplicon of the expected size in each case indicated the adjacent genes were part of polycistronic messengers (Figure 7). These results clearly show that in *S. metallicus *each couple of *copMA *genes was expressed in the form of transcriptional units, as reported in *F. acidarmanus *and S*. solfataricus *[18, 19]. The coexpression of gene pair's *copMA *may suggest a coordinated and dependent function for the respective encoded proteins. In the bacterium *E. hirae*, the metallochaperone CopZ (CopM in *S. metallicus*) fulfills a pivotal role in the mechanism of Cu homeostasis [45]. Thus, it was shown that this protein interacts directly with the Cu-ATPase CopB (CopA in *S. metallicus*), handing the Cu for subsequent removal. In this context, one might expect that proteins CopM1 and CopM2 from *S. metallicus *have a similar role to that described for *E. hirae*. Furthermore, quantitative RT-PCR experiments were carried out in order to determine differential expression of the *cop* genes in response to different Cu concentrations. The expression of *copA1*, *copA2*, *copT1 *and *copT2* was tested relative to the transcript levels of *rRNA 16S* gene since its levels were not significantly affected in all assessed conditions (data not shown). As shown in Figure 8(a), *copA2* and *copA1 *transcript levels were found to be concomitantly increased with the increasing Cu concentrations present in the medium. Higher transcripts levels of *copA1* were seen in all tested conditions when compared with *copA2* levels. *copA1 *transcript levels were found to be 32.5-fold up-regulated when comparing 50 mM Cu condition versuscontrol (absence of metal), whereas* copA2* transcript levels showed an increment of only 17.5-fold (Figure 8(a)). The finding that Cu-ATPases mRNA levels were significantly increased in response to Cu ions exposure suggests that the transport system may operate for Cu efflux in *S. metallicus*. 

Moreover, *copA2* and *copA1* gene expression was quantified when *S. metallicus* was grown using chalcopyrite (CuFeS_2_) ore as an oxidizable substrate (Figure 8(a)). Mineral oxidation mediated by the microorganism generates a progressive increase in Cu ions concentration in the medium. To find out whether the amount of solubilized Cu induced the expression of the Cu-ATPases genes, total RNA was extracted from a *S. metallicus* culture grown to late exponential phase and in the presence of 1% CuFeS_2_. It was clear that an up-regulation of the Cu-ATPases encoding genes also took place when *S. metallicus* was grown in the presence of CuFeS_2_ (Figure 8(a)). *copA1* transcript levels increased 14-fold compared with the control condition in the absence of Cu, while *copA2* gene expression showed an increase of 7.6 fold. Along with this, by means of atomic absorption spectrometry (AAS) analysis, an overall amount of solubilized Cu ions (Cu^2+^/Cu^1+^) of 14.4 ± 2.1 mM was determined to be present in the medium, indicating that *copA2* and *copA1* gene expression was most likely in response to the Cu present in the culture due to CuFeS_2_ microbial-solubilization.

The finding that *copA1* was highly expressed compared with *copA2 *in all tested conditions may suggest a possible physiological hierarchy between the two ATPases when overcoming either Cu or Cd stress. In this regard, by means of a genetic approach it was demonstrated in *S. solfataricus* that while CopA was an effective Cu efflux transporter at low and high Cu concentrations, the other Cu-ATPase (CopB) only appeared to be a low-affinity Cu export ATPase [68]. Moreover, by using a *M. sedula copA* deletion mutant it was demonstrated that this strain compromised metal resistance and consequently abolished chalcopyrite oxidation [22], highlighting the role of Cu detoxification mechanisms during a given bioleaching process. Our attempts to show the functionality of CopA1 and CopA2 from *S. metallicus* by using *E. coli* as a heterologous host were not successful. Apparently, the overexpression of these ORFs had a toxic effect on *E. coli* that compromised its viability. Although gene disruption tools have not been yet developed for *S. metallicus* the functional role of both *copTMA *locimight be further studied by cross-species complementation of a copper sensitive *S. solfataricus copR *mutant as it was described by Maezato et al. [22]. It will be of great interest to establish in future studies the possible functionality of the isolated putative transporters from *S. metallicus.*

Likewise, quantitative RT-PCR experiments showed that *copT1* transcript levels increased concomitantly with increasing Cu concentrations, whereas *copT2* showed relatively similar transcripts levels, most likely indicating a constitutive expression profile (Figure 8(b)). Furthermore, whereas *copT1* transcript levels were found to be increased 3.6 fold in CuFeS_2_ grown cultures, *copT2* levels remained unchanged in comparison with the control in the absence of Cu (Figure 8(b)). The results obtained for *S. metallicus copT2* gene expression are consistent with those reported for *S. solfataricus*. CopT transcriptional regulator has been proposed to function as a repressor in *S. solfataricus*, showing a constitutive expression that in the presence of Cu loses its affinity for the promoter region of *copMA* allowing the expression of this polycistron [19]. In contrast, the increased transcripts levels of both *copA1* and *copT1* concomitant with higher Cu concentrations in the environment suggest that the putative transcriptional regulator CopT1 may act by activating both *copMA1* and probably itself (Figure 8). Regarding this, it was recently reported that CopR (CopT in *S. metallicus*) from *S. solfataricus* strain 98/2 acts as an activator of *copT* (*copM* in *S. metallicus*) and *copA* expression [69]. Nevertheless, additional experiments would be required to test this possibility in *S. metallicus*.

## 4. Concluding Remarks

We previously reported that *S. metallicus* resists extremely high Cu concentrations, which was mediated to some extent by the use of a possible metal resistance system based on inorganic polyphosphate hydrolysis and consequently Cu-PO_4_^2−^efflux [15, 29, 30]. Here we have addressed the question whether this microorganism coded for other determinants that might help to explain its high heavy metal tolerance. As the genomic sequence of this microorganism is not yet available, we jointly used CODEHOP-PCR and genome walking approaches and were able to establish the occurrence of two nonidentical homologous *cop* loci into the genome of *S. metallicus* (*cop1* and *cop2*). Each *cop* locus codes for an archaeal transcriptional regulator (CopT), a metal-binding chaperone (CopM) and a Cu-transporting P-type ATPase (CopA). High levels of the polycistronic mRNAs*copMA* of each *cop* locus were observed after treatment with either Cu or Cd, suggesting that the encoded ATPases efflux heavy metals out in order to detoxify the intracellular environment. Altogether, previous reports and the results obtained in this study allow us to suggest that some key elements that may explain the high resistance to Cu in *S. metallicus* is the duplication of the Cu resistance *cop *genes, a defensive response to stress and a polyP-based accumulation mechanism. In the case of Cd, although some similar stress responses were observed, whether comparable Cu-responsive elements also participate in Cd responses remains to be seen. 

## Supplementary Material

CODEHOP-based PCR details for the amplification of the putative genes *copA* and *copM* from *S. metallicus* are provided in the supplementary material.

## Figures and Tables

**Table 1:** Oligonucleotides.

Name	Sequence	Description

copA_cdegF	5′-gatgtagtaatagtaaaaactggagaaataataccngcngaygg	CODEHOP-PCR
copA_cdegR	5′-tcatcagcaaaattagaagaatctccngtngcdat	CODEHOP-PCR
copT_cdegF1	5′-ctcaaatagaatataaagtattacaaatgttaaaagargaywsnmg	CODEHOP-PCR
copT_cdegR1	5′-ggattaccatttatttcatttccacartartcrca	CODEHOP-PCR
copT_cdegR2	5′-cttatcatattcataaatcttccatctattaatttrtarcaytc	CODEHOP-PCR
copT_degF1	5′-gartgytayaarctnat	DOP-PCR
copT_degR1	5′-atnagyttrtarcayct	DOP-PCR
copM_degF	5′-gayccngtntgyggnatgga	DOP-PCR
copM_degR	5′-ccnggnttyccntacgg	DOP-PCR
AdaptF2	5′-cacgcgtcgactagtactagctt	Genome Walking
SP1copA1_3′	5′-aaggatgagggggaccttatgg	Genome Walking
SP2copA1_3′	5′-ggagataagaaatggggtaaaagag	Genome Walking
SP1copA1_5′	5′-tgataccatcatggaacctgtcag	Genome Walking
SP2copA1_5′	5′-tcctccacaatcccatccgctg	Genome Walking
SP1copA1_5′_2	5′-gattgtagctaagttaacctcggcctcg	Genome Walking
SP2copA1_5′_2	5′-cttctcaccctcagtctggttgg	Genome Walking
SP1copA2_3′	5′-gaaagaggaatatatgcaagggtaaacgg	Genome Walking
SP2copA2_3′	5′-gtgttaatgggagagctggaggg	Genome Walking
SP1copA2_5′	5′-cttctctgtggcaacatcataaccagcc	Genome Walking
SP2copA2_5′	5′-acgcatgtggcgcaatgcattcc	Genome Walking
SP1copT1_5′	5′-cattcctcgcaccagcttgcacactctc	Genome Walking
SP1copT2_5′	5′-cctatgaatactagatcttttccctgaac	Genome Walking
SP2copT2_5′	5′-aacagcttataacactcgtcactttggc	Genome Walking
copM2_RT_F	5′-gatgaaaaaagccaatataagac	RT-PCR
copA2_Rv	5′-gaacactaactaacatcgcc	RT-PCR
copM1_RT_Fw	5′-ctatcgtttttgttccgaagcttg	RT-PCR
copA1_Rv	5′-cagcagcaagaacagagacgcc	RT-PCR
*SM16Sf	5′-acgctctaaaaaggcgtgggaata	RT-qPCR
*SM16Sr	5′-ttgagctcggggtctttaagcagtg	RT-qPCR
copA1Sm_qRT_F1	5′-gctaaggtaatagagagcgg	RT-qPCR
copA1Sm_qRT_R1	5′-tgaacaggaatggacagg	RT-qPCR
copA2Sm_qRT_F	5′-tgtgcttgtctccttagcgt	RT-qPCR
copA2Sm_qRT_R	5′-actcttccgtctttcggagt	RT-qPCR
copT1Sm_qRT_F	5′-tgtaggagagtgtgcaagct	RT-qPCRl
copT1Sm_qRT_R	5′-tcgcaagtgagggttatggt	RT-qPCR
copT2Sm_qRT_F	5′-gtgttacggagcttgca	RT-qPCR
copT2Sm_qRT_R	5′-acactcgtcactttggc	RT-qPCR

All oligonucleotides were synthesized by Invitrogen. *Oligonucleotides designed by Bathe and Norris [39].

**Table 2:** Proteins upregulated in *S. metallicus* cells exposed to 100 mM Cu or 1 mM Cd.

Spot	Molecular weight (kDa)	Cu induction levels	Putative function	Microorganism related	Accesion number
1^†^	26.6	∞	Proteasome subunit	*S. solfataricus *	AAK41034
3^†^	70.1	∞	Ferredoxin oxidoreductase	*S. solfataricus *	AAK42926
4^†^	42.9	∞	Alcohol dehydrogenase	*S. solfataricus *	AAK43154
5	35	2.6	Hypothetical protein	*S. acidocaldarius *	YP_254976
6	27	∞	Hypothetical protein	*P. aerophilum *	NP_559889
9^†^	57	∞	Phosphoglycerate dehydrogenase	*M. thermautotrophicus *	AAB85466
13^†^	27	3.4	Unknown	—	—
14	28	∞	Unknown	—	—
15	35	5.5	Unknown	—	—
20^†^	46.1	∞	Glutamate dehydrogenase	*S. solfataricus *	AAK42230
21^†^	45	∞	Hypothetical protein	*M. acetivorans *	NP_618380.1
22	49	∞	Hypothetical protein	*P. torridus *	YP_022807
23^†^	51	∞	ATP synthase subunit B	*S. solfataricus *	AAK40880
24	58.5	∞	HSP60 subunit	*S. shibatae *	AAG37273.1
30^†^	48	∞	Unknown	—	—
31	16	ND	Unknown	—	—
32	14.4	ND	Putative transcription regulator	*S. solfataricus *	AAK40413
33	13.8	ND	Transcription factor nusA	*S. solfataricus *	AAK40563

Spots refer to those numbered in Figure 2. Proteins in Figure 2 which are not included in this table were not subjected to sequencing due to their low intensities in the gels.

^†^Spot up-regulated in cells exposed to Cu and Cd.

ND: not determined.

Infinity symbol indicates that proteins were expressed only in presence of copper or cadmium.

*M. thermoautotrophicus*: *Methanobacterium thermoautotrophicus*; *M. acetivorans*: *Methanosarcina acetivorans*; *S. shibatae*: *Sulfolobus shibatae*; *P. torridus*: *Picrophilus torridus*; *P. aerophilum*: *Pyrobaculum aerophilum*; *S. acidocaldarius*: *Sulfolobus acidocaldarius*.

**Table 3:** Sequence identity comparison of *S. metallicus *Cop proteins to those in other *Sulfolobales*.

*S. metallicus* protein	% Identity to homologue in other *Sulfolobales *			
	*S. solfataricus *P2	*S. acidocaldarius *	*S. tokodaii *	*M. sedula *
CopA1	**51**	43	44	45
CopA2	**88**	46	49	50
CopM1	63	69	**71**	**71**
CopM2	**89**	72	70	77
CopT1	**36**	27	30	32
CopT2*	**90**	52	52	53

Boldface type indicates to which organism the *S. metallicus* homologue shows the highest sequence identity.

*Refers to the analysis obtained using the uncompleted amino acid sequence of CopT2, which represents ~90% in length when compared with *S. solfataricus* CopT sequence.
