# Supplementary figures and images for: Molecular Characterization of Copper and Cadmium Resistance Determinants in the Biomining Thermoacidophilic Archaeon Sulfolobus metallicus
Source: Archaea. 2013 Feb 24;2013:289236. doi: 10.1155/2013/289236 (PMC3595675; doi:10.1155/2013/289236)

## Slide 1
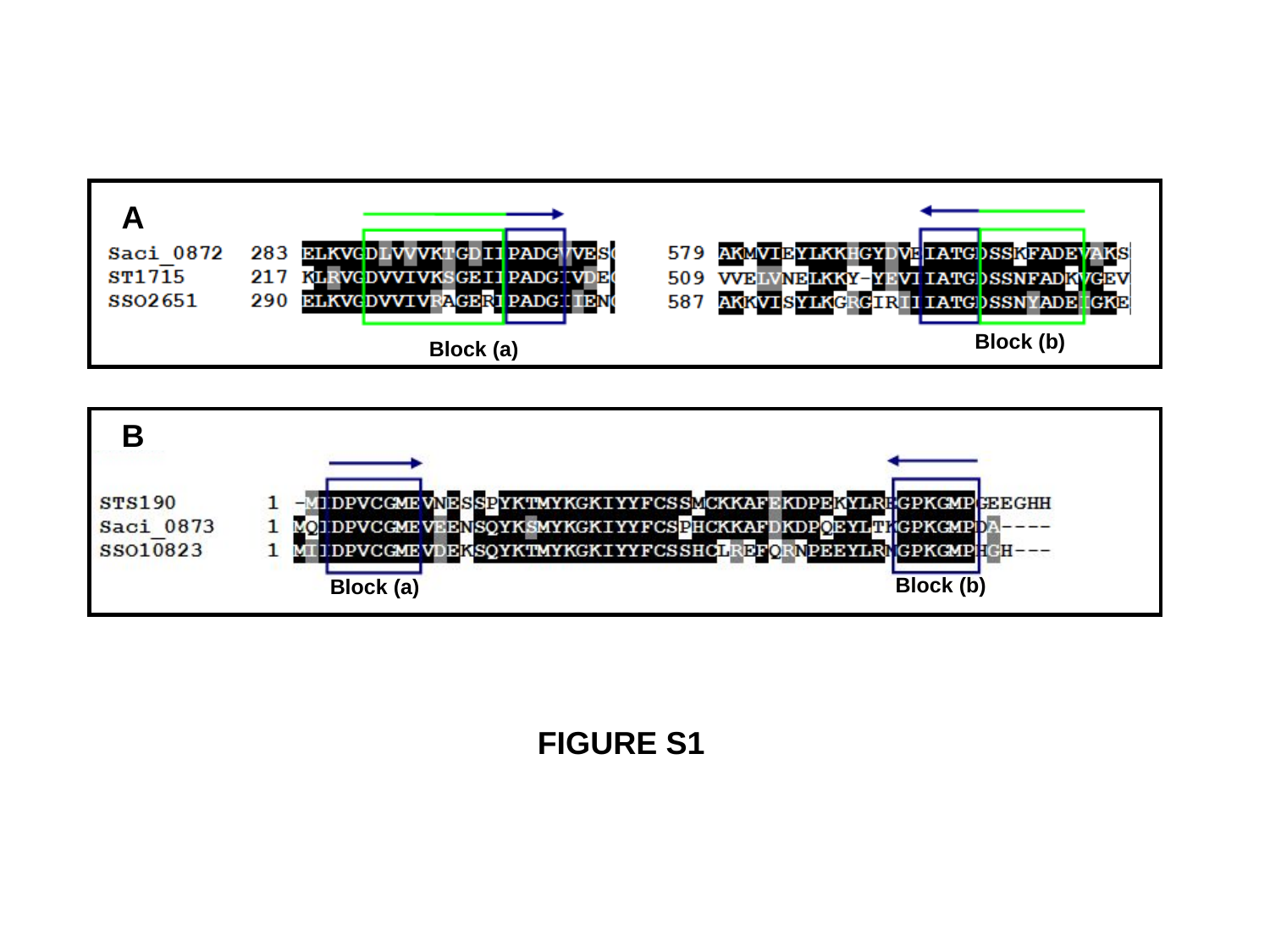

A
Block (b)
Block (a)
B
Block (b)
Block (a)
FIGURE S1

Supplement: Supplementary file 1 — CODEHOP-based PCR details for the amplification of the putative genes copA and copM from S. metallicus are provided in the supplementary material. [file 289236.f1.ppt]

## Slide 1
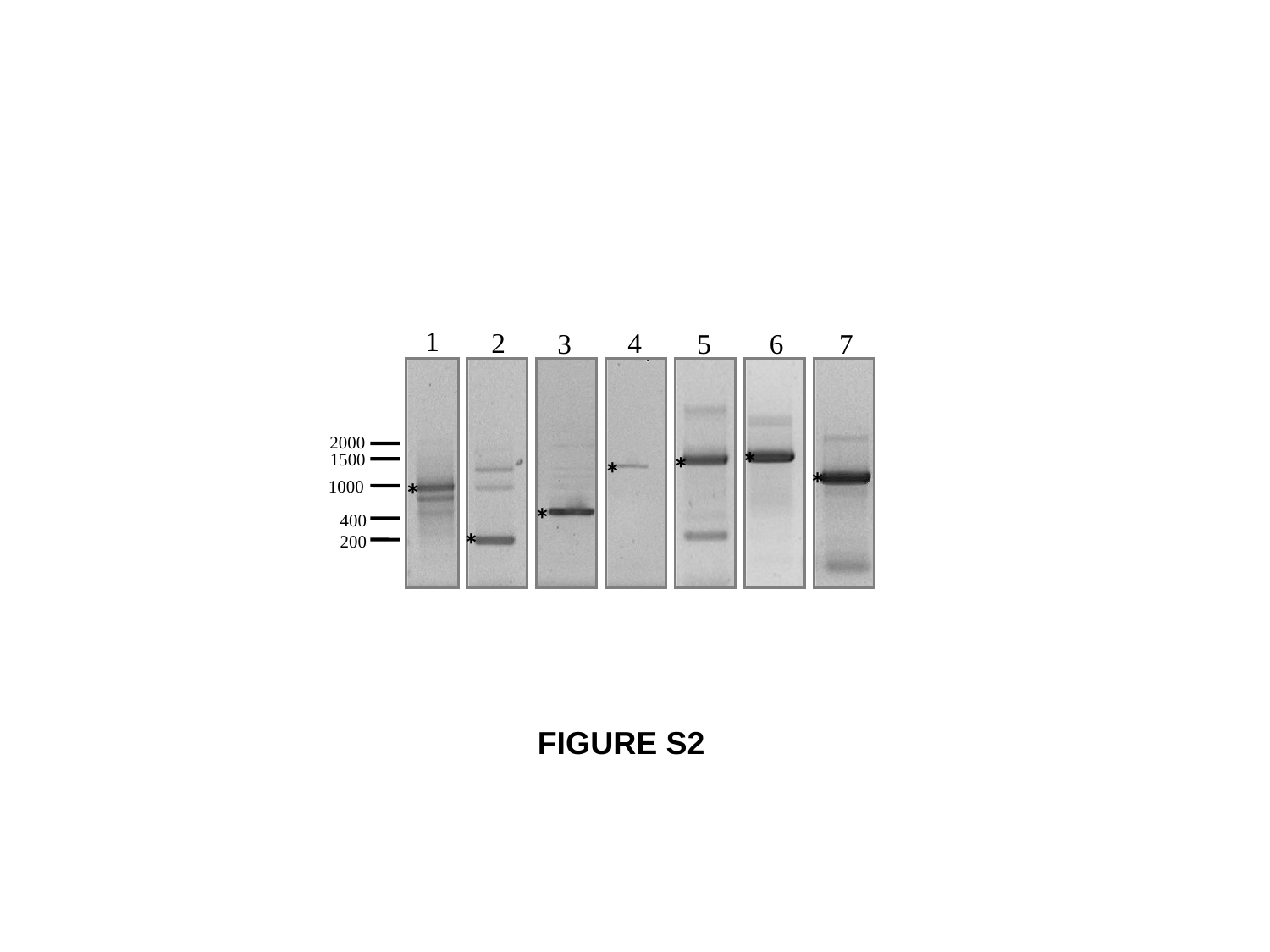

1
4
2
3
5
6
7
2000
*
1500
*
*
*
1000
*
*
400
*
200
FIGURE S2

Supplement: Supplementary file 2 [file 289236.f2.ppt]
